# Comparative assessment of efficacy and safety of approved oral therapies for overactive bladder: a systematic review and network meta-analysis

**DOI:** 10.1590/S1677-5538.IBJU.2023.0158

**Published:** 2023-06-30

**Authors:** Wenjuan He, Guangliang Huang, Wenyan Cui, Yunfei Tian, Qian Sun, Xiaojuan Zhao, Yonghong Zhao, Dan Li, Xiuju Liu

**Affiliations:** 1 Second Hospital of HeBei Medical University Department of Pharmacy Shijiazhuang Hebei China Department of Pharmacy, the Second Hospital of HeBei Medical University, Shijiazhuang (Hebei), China;; 2 HeBei Medical University Department of Clinical Pharmacy Shijiazhuang Hebei China Department of Clinical Pharmacy, HeBei Medical University, Shijiazhuang (Hebei), China;; 3 University of Hong Kong Department of psychology Hong Kong China Department of psychology, the University of Hong Kong, Hong Kong, China

**Keywords:** Urinary Bladder, Overactive, Safety, Network Meta-Analysis

## Abstract

**Purpose:**

To compare the effectiveness and safety of marketed oral drugs for overactive bladder based on a systematic review and network meta-analysis approach.

**Methods:**

Pubmed, Embase, Web of Science, and the Cochrane Register of Clinical Trials databases were systematically searched. The search time frame was from database creation to June 2, 2022. Randomized controlled double-blind trials of oral medication for overactive bladder were screened against the protocol's entry criteria. Trials were evaluated for quality using the Cochrane Risk of Bias Assessment Tool, and data were statistically analyzed using Stata 16.0 software.

**Result:**

A total of 60 randomized controlled double-blind clinical trials were included involving 50,333 subjects. Solifenacin 10mg was the most effective in mean daily micturitions and incontinence episodes, solifenacin 5/10mg in mean daily urinary urgency episodes and nocturia episodes, fesoterodine 8mg in urgency incontinence episodes/d and oxybutynin 5mg in voided volume/micturition. In terms of safety, solifenacin 5mg, ER-tolterodine 4mg, mirabegron, vibegron and ER-oxybutynin 10mg all showed a better incidence of dry mouth, fesoterodine 4mg, ER-oxybutynin 10mg, tolterodine 2mg, and vibegron in the incidence of constipation. Compared to placebo, imidafenacin 0.1mg showed a significantly increased incidence in hypertension, solifenacin 10mg in urinary tract infection, fesoterodine 4/8mg and darifenacin 15mg in headache.

**Conclusion:**

Solifenacin showed better efficacy. For safety, most anticholinergic drugs were more likely to cause dry mouth and constipation, lower doses were better tolerated. The choice of drugs should be tailored to the patient's specific situation to find the best balance between efficacy and safety.

## INTRODUCTION

Overactive bladder (OAB) consists of four closely related symptoms: urgency, frequency, urge urinary incontinence (UUI) and nocturia, which have no significant impact on the patient's life safety but seriously reduce the quality of life. Studies have shown (1) that OAB can have varying degrees of impact on six aspects of daily life: recreational life, psychological problems, isolation, sexual desire, and work efficiency, causing a heavy economic burden on patients and society. The prevalence of OAB is high, ranging from 7% to 27% in men and 9% to 43% in women, and the prevalence of OAB increases with age (2, 3). However, the pathophysiological mechanisms involved in the symptoms of OAB syndrome are varied and treatment is difficult (4). For this reason, more and more scholars have been conducting research on the pathogenesis of OAB from different perspectives in recent years and are constantly exploring new treatments for OAB. Treatment options for OAB are divided by “lines of therapy” based on levels of invasiveness. Lifestyle modification and pelvic floor physical therapy are the tenets of the first line of therapy. Second line therapy consists of drug therapy with anticholinergics and/or beta-3 agonists. Third line therapies include intravesical botulinum toxin injection, sacral neuromodulation, and percutaneous tibial nerve stimulation (5, 6).

For decades, antimuscarinics such as tolterodine (TOL) and solifenacin (SOL) have been the main pharmacological treatment for OAB, but their lack of bladder specificity has led to a high incidence of adverse events such as dry mouth and constipation, ultimately limiting their effectiveness. In recent years, β3-adrenoceptor agonists, which are highly selective, have been developed as a potential treatment for OAB. Pharmacological assays have shown that β3-adrenoceptor agonists participate in beta adrenergic-mediated bladder relaxation, thus exerting their effect (5). They have been shown to be effective and well tolerated (7, 8).

Different treatment modalities have their advantages and limitations, and it is essential to choose the right treatment modality for the specific patient in clinical practice. The wide choice of drugs available for OAB treatment and the lack of head-to-head clinical trials between drugs has led to controversy over the best drug choice. Given that one previously published study (9) had too many drug doses (including unapproved doses) grouped together, and the outcome indicators were not combined in a reasonable manner, the potential for bias is too high and the robustness of the final study results is questionable. Therefore, this study proposes to conduct a precise network meta-analysis of approved oral drugs, including only oral drugs with approved dosages and only outcome indicators with the same observation period, in order to reduce the heterogeneity of the introduced studies and provide a basis for the selection of therapeutic drugs in clinical practice.

## MATERIALS AND METHODS

The software involved in this study included EndNote X8 (literature management and article writing) (Thomson Research Soft), Excel 2019 (data extraction and collation) (Microsoft Office), Review Manager 5.3 (methodological quality evaluation) (The Cochrane Collaboration, Copenhagen), and Stata 16.0 (network meta-analysis [NMA], heterogeneity assessment and inconsistency testing, surface under the cumulative ranking curve [SUCRA] plots) (Stata Corporation). The study was written according to the NMA extension for Priority Reporting Entry for Systematic Evaluation and Meta-Analysis (PRISMA). This study is registered with PR0SPERO (registration number CRD42021233959).

### Search strategies

Two reviewers searched independently in the following database: PubMed, Embase, Web of Science and Cochrane Library. Both mesh terms and free terms were used in the search. Details of search strategies are provided in Supplementary Table-1 (see Page 1).

**Table 1:** Basic Characteristics of Included Study.

Study trial number	Study design	Country	Intervention	Population mean age	Female (%)	Numbers of patients (n)	Treatment duration (weeks)
Yoshida et al. (37) 2018 No. JapicCTI- 152936	Phase IIb, RCT, double-blind, multicenter	Japanese	VIB 50mg, qd	58.0 ± 11.8	334 (90.3)	370	aged ≥ 20 years, patients experiencing OAB symptoms for ≥ 6 months
			VIB 100mg, qd	58.7 ± 11.1	330 (89.7)	368	
			PBO	58.9 ± 11.8	333 (90.2)	369	
			IMI 0.1mg, bid	59.7 ± 12.4	105 (89.7)	117	
Yamaguchi et al. (30) 2014a NCT00966004	Phase III, RCT, double-blind, multicenter	Japanese	MIR 50mg, qd	58.3 ± 13.88	58 (15.7)	379	aged ≥ 20 years, patients experiencing OAB symptoms for ≥ 24 weeks
			PBO	58.2 ± 14.18	58 (15.8)	379	
			TER 4 mg, qd	58.3 ± 13.96	64 (17.4)	375	
Yamaguchi et al. (38) 2014b NCT00527033	Phase II, RCT, double-blind, multicenter	Japanese	MIR 50mg, qd	56.2 ± 13.59	31 (14.9)	208	aged ≥ 20 years, patients experiencing OAB symptoms for ≥ 24 weeks
			PBO	55.7 ± 12.89	42 (19.9)	212	
Staskin et al. (43) 2020 NCT03492281	Phase III, RCT, double-blind, multicenter	Multinational	VIB 75mg, qd	63.0 ± 18.0	449 (85.4)	545	aged ≥ 18 years, patients experiencing OAB symptoms for ≥ 3 months
			PBO	61.0 ± 16.0	445 (85.6)	540	
			TER 4 mg, qd	61.0 ± 17.0	352 (84.4)	430	
Shin et al. (55) 2019	Phase IV, RCT, double-blind, multicenter	Korea	MIR 50mg, qd	66.40 ± 9.51	310 (100)	310	aged ≥ 20 years, patients experiencing OAB symptoms for ≥ 12 weeks
			PBO	65.23 ± 10.00	154 (100)	154	
Nitti et al. (40) 2013 NCT00662909	Phase III, RCT, double-blind, multicenter	United States and Canada	MIR 50mg, qd	59.2 ± 13.5	120 (27.1)	442	aged ≥ 18 years, patients experiencing OAB symptoms for ≥ 3 months
			PBO	60.1 ± 13.8	108 (23.8)	453	
Herschorn et al. (41) 2013 NCT00912964	Phase III, RCT, double-blind, multicenter	Europe and North America	MIR 50mg, qd	60.3 ± 12.22	137 (31.1)	440	aged ≥ 18 years, patients experiencing OAB symptoms for ≥ 3 months
			PBO	58.2 ± 13.73	132 (30.5)	433	
Kuo et al. (70) 2015 NCT01043666	Phase III, RCT, double-blind, multicenter	Taiwan, Korea, China, and India	MIR 50mg, qd	54.3 ± 14.21	110 (32.5)	366	aged ≥ 18 years, patients experiencing OAB symptoms for ≥ 3 months
			PBO	55.3 ± 13.63	98 (30.3)	366	
			TER 4 mg, qd	53.9 ± 14.50	120 (36.0)	371	
Khullar et al. (44) 2013 NCT00689104	Phase III, RCT, double-blind, multicenter	European–Australian	MIR 50mg, qd	59.1 ± 12.36	136 (27.6)	493	aged ≥ 18 years, patients experiencing OAB symptoms for ≥ 3 months
			MIR 100mg, qd	59.0 ± 12.71	141 (28.4)	496	
			PBO	59.2 ± 12.30	138 (27.9)	494	
			TER 4 mg, qd	59.1 ± 12.89	134(27.1)	495	aged ≥ 18 years, patients experiencing symptoms of wet OAB for ≥ 3 months
Herschorn et al. (42) 2017 NCT01972841	Phase III, RCT, double-blind, multicenter	Multinational (42 countries)	MIR 50mg, qd	56.7 ± 13.3	99 (23.5)	422	
			PBO	57.9±13.0	102 (23.8)	429	
Chapple et al. (71) 2013 NCT00337090	Phase II, RCT, double-blind, multicenter	Multinational	MIR 50mg, qd	56.9 ± 12.5	18 (10.8)	169	aged ≥ 18 years, patients experiencing symptoms of OAB for ≥ 3 months
			PBO	57.1 ± 12.9	15 (9.0)	169	
			TER 4 mg, qd	56.6 ± 12.8	16 (18.8	85	
Herschorn et al. (42) 2017 NCT01314872	Phase IIb, RCT, double-blind, multicenter	Multinational (18 countries)	MIR 50mg, qd	60.3 ± 8.7	129 (86.0)	150	aged ≥ 18 years and ≤ 75years, patients experiencing symptoms of OAB for ≥ 3 months
			PBO	57.8 ± 9.5	185 (90.2)	205	
			TER 4 mg, qd	58.5 ± 9.6	231 (89.9)	257	
Armstrong et al. (58) 2005	RCT, double-blind, multicenter	Multicenter	ER-OXY 10mg, qd	60 (18–92)	100%	391	aged ≥ 18 years, patients experiencing symptoms
			TER 4 mg, qd	60 (18–92)	100%	399	
Cardozo et al. (59) 2004	RCT, double-blind, multicenter	Multinational	SOL 5mg, qd	55.4 (13.8)	237 (82.9)	286	aged ≥ 18 years, patients experiencing symptoms of OAB for ≥ 3 months
			SOL 10mg, qd	55.9 (14.2)	238 (82.1)	290	
			PBO	56.1 (13.3)	227 (80.8)	281	
Chapple et al. (57) 2007a	RCT, double-blind, multicenter	Multinational	DAR 7.5/15 mg, qd	72 ± 5 (64–89)	206 (77.4)	266	aged ≥ 65 years with symptoms of OAB for at least 6 months
			PBO	73 ± 5 (64–87)	100 (75.2)	133	
Chapple et al. (60) 2014 NCT01302067	RCT, double-blind, multicenter	Multinational	FES 4 mg, qd	59.8 (21–94)	647 (82)	790	aged ≥ 18 years with OAB symptoms for ≥ 6 months
			FES 8 mg, qd	58.8 (18–89)	627 (80)	779	
			PBO	59.6 (19–85)	316 (82)	386	
Chapple et al. (15) 2007b	Phase III, RCT, double-blind, multicenter	Multinational	TER 4 mg, qd	57.7±14.6	226 (78)	290	aged ≥ 18 years with OAB symptoms for ≥ 6 months
			FES 8 mg, qd	55.6 ± 14.1	223 (82)	272	
			FES 4 mg, qd	57.1 ± 13.2	232 (81)	287	
			PBO	56.0±13.7	229 (81)	283	
Chapple et al. (61) 2005	RCT, double-blind, multicenter	European	SOL 5 mg/10mg, qd	56.5	493 (85.3%)	578	aged ≥ 18 years, patients experiencing OAB symptoms for ≥ 3 months
			TER 4 mg, qd	56.4	529 (88.3%)	599	
Chapple et al. (16) 2004	Phase IIIa, RCT, double-blind, multicenter	Multinational	SOL 5 mg, qd	58.1 (13.4)	194 (72.9)	266	aged ≥ 18 years, patients experiencing OAB symptoms for ≥ 3 months
			SOL 10 mg, qd	57.2 (13.4)	188 (71.2)	264	
			TER 2mg bid	56.9 (12.8)	200 (80.0)	250	
			PBO	57.8 (13.7)	193 (76.3)	253	
Choo et al. (17) 2008 NCT00189800	RCT, double-blind, multicenter	Korea	SOL 5 mg, qd	53.07 10.52	90 (84.11)	107	aged ≥ 18 years, patients experiencing OAB symptoms for ≥ 3 months
			SOL 10 mg, qd	52.65 (12.71	83 (74.77)	111	
			TOL 2 mg, bid	53.05 (12.19	88 (79.28)	111	
Chu et al. (20) 2009	Phase III, RCT, double-blind, multicenter	United States	SOL 10 mg, qd	59 (14)	272 (80.0)	340	aged ≥ 18 years with a diagnosis of OAB made by an investigator based on symptoms
			PBO	58 (13)	277 (83.4)	332	
Chua et al. (18) 2018 NCT01486706	RCT, double-blind, single center	Philippines	SOL 5 mg/10mg, qd	57.2 (9.36)	24 (77%)	31	18–79 years old, patients who are ambulatory, with defined history of OAB symptoms for ≥ 3 months
			PBO	53.9 (12.14)	23 (72%)	32	
Chuang et al. (19) 2020	RCT, double-blind, multicenter	Taiwan	IMI 0.1 mg,bid	59.84	23 (31.5%)	73	patients ≥ 20 years of age, with OAB symptoms for ≥ 3 months
			PBO	59.33	19 (48.7%)	39	
Diokno et al. (62) 2003	RCT, double-blind, multicenter	US	OXY 10 mg, qd	(23, 92)	100%	391	Women with OAB symptoms, aged 18 years and older
			TER 4mg, qd	(18, 85)	100%	399	
Dmochowski et al. (21) 2010	RCT, double-blind, multicenter	US	FES 4mg/8mg, qd	59.7 (13.7)	364 (83)	438	Aged ≥ 18 years patients experiencing OAB symptoms for ≥ 3 months
			PBO	60.1 (12.9)	368 (83)	445	
Dmochowski et al. (22) 2008	Phase III, RCT, double-blind, multicenter	US	TRO 60mg, qd	61.2 ± 0.7	230 (82.1)	280	Subjects aged 18 years or older with OAB of 6 months or longer duration
			PBO	58.4 ± 0.7	249 (87.7)	284	
Drutz et al. (14) 1999	RCT, double-blind, multicenter	United States and Canada	TOL 2 mg, bid	63.0 (31–88)	88 (81)	109	aged ≥ 18 years, patients experiencing OAB
			OXY 5 mg, tid	66.3 (23–91)	81 (72)	112	
			PBO	62.1 (26–87)	45 (80)	56	
DuBeau et al. (23) 2014 NCT00928070	RCT, double-blind, multicenter	US	FES 4mg/8mg, qd	74.8 (65- 91)	100%	103	65 years old or older with OAB symptoms for 3 or more months
			PBO	75.3 (65-90)	100%	77	
Ercan et al. (63) 2015	RCT, single center	Turkey	SOL 5 mg, qd	58.9 ± 11.5	UK	60	patients diagnosed with OAB
			FES 4 mg, qd	58.1 ± 10.258.1	UK	59	
Ginsberg et al. (64) 2013	RCT, double-blind, multicenter	Multinational	FES 4mg/8mg, qd	59.8 (14.3) 57.5 (13.0)	1374 (84)	1639	≥ 18 years old, had self-reported OAB symptoms for ≥ 3 months
			TER 4mg, qd	60.8 (14.1) 57.8 (13.4)	1382 (83)	1657	
			PBO	61.8 (13.9) 58.5 (13.2)	679 (84)	812	
Gotoh et al. (24) 2011	Phase III, RCT, double-blind, multicenter	Japan	PRO 20 mg, qd	56.6 (13.6)	216 (76.1)	284	≥ 20 years old with OAB symptoms for at least 12 weeks
			PBO	58.7 (14.1)	207 (76.7)	270	
Govier et al. (30) 2010	Phase III, RCT, double-blind, multicenter	US	SOL 10 mg, qd	60 ± 13	261 (82)	318	Aged ≥ 18 years with OAB symptoms
			PBO	59 ± 13	259 (82)	316	
Herschorn et al. (41) 2013 NCT01767519	Phase IIIb, RCT, double-blind, multicenter	North America and Europe	SOL 5 mg/10mg, qd	61.4 ± 12.8	134 (88.7)	151	Adults with symptoms of patients diagnosed OAB for ≥ 6 months
			PBO	62.9 ± 11.8	51 (85.0)	60	
Homma et al. (53) 2003	RCT, double-blind, multicenter	Japanand Korea	TER 4 mg, qd	61.2 (11.8)	162 (68)	239	aged ≥ 20 years with symptoms of OAB for ≥ 6 months
			OXY 3 mg, qd	57.9 (12.5)	177 (73)	244	
			PBO	58.4 (14.0)	84 (69)	122	
Homma et al. (25) 2009	Phase III, RCT, double-blind, multicenter	Japan	IMI 0.1 mg, bid	57.7 (12.7)	278 (87.4%)	324	≥ 20 years, who had OAB symptoms
			PRO 20 mg, qd	59.8 (11.9)	257 (84.3%)	310	
			PBO	58.0 (13.5)	125 (87.4%)	147	
Homma et al. (26) 2008	Phase II, RCT, double-blind, multicenter	Japan	IMI 0.1 mg, bid	64.5 (13.5)	63 (67.7)	93	≥ 20 years, who had OAB symptoms
			PBO	61.9 (11.8)	69 (72.6)	95	
Kaplan et al. (45) 2014 NCT01302054	RCT, double-blind, multicenter	Europe, North America, Asia, and Africa	FES 4mg/8mg, qd	57.3 (13.4)	253 (82)	308	aged ≥ 18 years, self-reported OAB symptoms for ≥ 6 months
			PBO	58.2 (13.2)	244 (81)	301	
Karram et al. (32) 2009 NCT00454896	Phase IIIb, RCT, double-blind, multicenter	USA	SOL 5 mg/10mg	57	84.20%	372	age 18 or older, OAB for at least 3 months
			PBO	57	84.20%	367	
Lee et al. (28) 2013 NCT01578304	Phase IV, RCT, double-blind, multicenter	Korean	IMI 0.1 m, bid	57.94 ± 10.81	57.94 ± 10.81	104	aged ≥ 20 years, with OAB symptom for ≥ 3 months
			FES 4 mg, qd	57.63 ± 12.63	57.63 ±12.63	102	
Nitti et al. (46) 2007	Phase III, RCT, double-blind, multicenter	US	FES 4 mg, qd	59 (21–85)	213 (76)	282	18 years or older with OAB syndrome for 6 months or greater
			FES 8 mg, qd	59 (23–91)	218 (78)	279	
			PBO	59 (24–88)	200 (74)	271	
Park et al. (47) 2014	Phase III, RCT, double-blind, multicenter	Korea	IMI 0.1 m, bid	58.31 ± 11.45	57 (85.07)	82	OAB patients aged ≥ 19 years for ≥ 3 months.
			PRO 20mg, qd	56.13 ± 11.29	55 (85.94)	80	
Rudy et al. (66) 2006	Phase III, RCT, double-blind, multicenter	US	TRO 40 mg, qd	61.1 ± 0.69	267 (81.2)	329	18 years or older with OAB symptoms for at least 6 months.
			PBO	61.0 ± 0.70	269 (81.8)	329	
Sand et al. (67) 2004	RCT, double-blind, multicenter	US	ER-OXY 10 mg, qd	58.4	100%	152	Participants with overactive bladder
			TOL 2mg, bid	58.8	100%	163	
Vardy et al. (33) 2009 NCT00573508	Phase IV, RCT, double-blind, multicenter	US	SOL 5 mg/10mg, qd	59 ± 13	306 (81)	377	(aged ≥ 18 years) were required to have OAB symptoms for ≥ 3 months
			PBO	60 ± 12	314 (84)	374	
Wagg et al. (34) 2013 NCT00798434	RCT, double-blind, multicenter	Multinational	FES 4mg/8mg, qd	72.6 ± 5.8	213 (54)	392	aged 65 and older with OAB symptoms for 3 months or longer
			PBO	72.8 ± 5.7	205 (52)	393	
Weiss et al. (50) 2013 NCT00911937	RCT, double-blind, multicenter	US	FES 4mg/8mg, qd	58.0 ± 14.7	313 (67.6)	463	age 18 years or older with self-reported OAB symptoms for 3 or more months
			PBO	57.5 ± 14.0	312 (65.8)	474	
Yamaguchi et al. (29) 2007	Phase III, RCT, double-blind, multicenter	Japan	SOL 5 mg, qd	60.4 (13.3)	318 (83.0)	398	aged ≥ 20 years and with symptoms of OAB reported for ≥ 6 months
			SOL 10 mg, qd	59.9 (13.0)	318 (85.7)	381	
			PRO 20 mg, qd	59.6 (13.6)	321 (83.6)	400	
			PBO	60.8 (12.5	333 (84.3)	405	
Yamaguchi et al. (27) 2011 NCT00561951	Phase II, RCT, double-blind, multicenter	Japan, Taiwan, Korea, and Hong Kong	FES 4 mg, qd	57.2 (14.2)	251 (78.4)	320	≥ 20 years of age; a medical history of OAB symptoms for ≥ 6 months
			FES 8 mg, qd	58.8 (13.4)	255 (81.5)	313	
			PBO	56.7 (13.5)	251 (78.9)	318	
Yamaguchi et al. (38) 2014b JapicCTI-101309	RCT, double-blind, multicenter	Japan	PRO 20 mg, qd	55.6 (12.5)	478 (85.5)	576	Age ≥ 20 years, OAB symptoms for ≥ 24 weeks
			PBO	56.2 (13.2)	344 (92.2)	381	
Zinner et al. (68) 2004	Phase III, RCT, double-blind, multicenter	US	TRO 20 mg, qd	63 ± 0.8	203 (77.5)	256	aged ≥ 18 years with a history of OAB for ≥ 6 months
			PBO	61.5 ±0.8	186 (71.3)	256	
Zinner et al. (69) 2006	RCT, double-blind, single center	US	DAR 15 mg, qd	59.1 (20–93)	185 (86.4)	214	aged ≥ 18 years with a history of OAB for ≥ 6 months
			PBO	59.1 (18–89)	198 (88.0	225	
Dmochowski et al. (54) 2003	RCT, double-blind, multicenter	UK	ER-TOL 4mg, qd	62.9[13.5]	117 (95.1)	123	at least 18 years of age taking current pharmacologic treatment for OAB
			PBO	64.5 [12.3]	109 (93.2)	117	
Haab et al. (72) 2004	RCT, double-blind, multicenter	Multinational	DAR 7.5 mg, qd	57.7 (22–88)	194 (84.7)	229	(aged 19–88 years, 85% female) who had suffered from symptoms of OAB for at least 6 months
			DAR 15 mg, qd	56.6 (24–81)	100 (87.0)	115	
			PBO	56.5 (19–81)	138 (84.1)	164	
Herschorn et al. (49) 2008 NCT00143377	RCT, double-blind, multicenter	Multinational	ER-TOL 4 mg, qd	58 (13)	290 (72)	408	aged ≥ 18 years with a history of OAB for ≥ 3 months
			PBO	57 (14)	143 (71)	204	
Hill et al. (73) 2006	RCT, double-blind, multicenter	Multinational	DAR 7.5 mg, qd	56.1 (23–88)	94 (87.04)	108	aged ≥ 18 years with a history of OAB for ≥ 6 months
			DAR 15 mg, qd	55.1 (24–82)	92 (85.98)	107	
			PBO	53.7 (21–85)	90 (82.57)	109	
Kaplan et al. (48) 2011 NCT00611026	RCT, double-blind, multicenter	Multinational	ER-TOL 4 mg, qd	58.1 (13.8)	818 (84)	960	(≥ 18 years) self-reported OAB symptoms for ≥ 3 months
			FES 4mg/8mg, qd	57.9 (13.5)	816 (85)	973	
			PBO	59.5 (13.2)	410 (86)	478	
Van Kerrebroeck et al. (35) 2001	RCT, double-blind, multicenter	Australasia, Europe and North America	ER-TOL 4 mg, qd	60 (20–89)	417(82.25)	507	aged ≥ 18 years with a history of OAB for ≥ 6 months
			TOL2 mg, bid	60 (22–92)	408(79.38)	514	
			PBO	61 (22–93)	410(80.71)	508	
Rogers et al. (51) 2008 NCT00143481	RCT, double-blind, multicenter	US	ER-TOL 4 mg, qd	49 (12)	100%	202	aged ≥ 18 years with OAB symptoms for ≥ 3 months
			PBO	47 (12)	100%	211	
Zinner et al. (36) 2002	RCT, double-blind, multicenter	Europe, United States, Canada, Australia, and New Zealand	ER-TOL 4 mg, qd	51 ± 10.5	417 (82.25)	507	aged ≥ 18 years with OAB symptoms for ≥ 6 months
			PBO	74 ± 6	410 (80.71	508	
Batista et al. (56) 2015	Phase III, RCT, double-blind, multicenter	Multinational	MIR 50 mg, qd	56.7 (14.3)	712 (76.1)	936	aged ≥ 18 years old, with symptoms of OAB for ≥ 3 months
			SOL 5 mg, qd	57.4 (13.6)	709 (75.9)	934	

Abbreviations: OXY = Oxybutynin; ER-OXY = Oxybutynin chloride extended-release; TOL = tolterodine; ER-TOL = extended-release tolterodine; SOL = solifenacin; CR-DAR = darifenacin extended-release; FES = fesoterodine; IMI = imidafenacin; PRO = propiverine; TRO = trospium chloride; VIB = vibegron; MIR = mirabegron; PBO = placebo

### Inclusion criteria

1. Study population: patients ≥18 years of age with a diagnosis of OAB according to symptoms or urodynamic studies.
2. Intervention: any drug approved for the treatment of OAB, or placebo as control, or another drug for the treatment of OAB as control.
3. Efficacy indicators: micturitions/d; incontinences/d; urgency episodes/d; urgency incontinences/d; nocturia episodes; mean voided volume/void.
4. Safety indicators: dry mouth; constipation; nasopharyngitis; hypertension; cardiovascular AEs; urinary tract infection.
5. Study type: randomized, controlled, double-blind trial with a follow-up period of ≥12 weeks.

### Exclusion criteria

Trials without any access to full text (eg, conference abstracts, etc.), with incomplete data, lack of relevant outcome indicators, data not publicly available and duplicate publications were excluded. Studies with non-oral antimuscarinic or intravesical administrations were also excluded.

### Literature screening and data extraction

1. Literature Screening: the literature was screened using EndNote X8 software to electronically check the literature retrieved from the systematic search and the manual search to eliminate duplicate literature. Then, two investigators independently read the titles and abstracts of the literature to exclude those that did not meet the inclusion criteria. After that, the remaining literature was read further in full to exclude those that did not meet the inclusion criteria, and the reasons for exclusion were recorded. Finally, both sides cross-checked the included literature and jointly decided on the inclusion of the literature, and in case of disagreement, a third investigator was consulted to decide on the inclusion of the literature.
2. Data extraction: data extraction was performed using Excel 2019 software, which included: authors and year of publication, sample size, interventions, baseline characteristics of the study population, and outcome indicators of the literature. Two researchers worked independently and discussed and resolved any disagreements or consulted a third researcher to decide. If incomplete information or disagreements were encountered in the literature study, the authors of the literature could be contacted for information.

### Methodological quality evaluation

The risk of bias was assessed in the included literature using the Cochrane Risk of Bias Assessment Tool (10) in Review Manager 5.3 software, including seven aspects: random sequence generation, allocation concealment, blinding of investigators and subjects, blinded evaluation of study outcomes, completeness of outcome data, and selective reporting of study results and other biases. For each study element, the investigator made a risk of bias assessment profile according to “low risk”, “high risk” and “unclear”.

## Statistical Analysis

We used the frequentist framework to perform a random effect network meta-analysis. The mean difference (MD) was used as an effect indicator for continuous variables, and odds ratio (OR) was used as an effect indicator for dichotomous variables. A 95% confidence interval (CI) was calculated for each effect size, and differences were considered statistically significant when P<0.05. Uncertainty in the effect of heterogeneity was defined as the inconsistency between the CI of the relative treatment effect and its prediction interval (11). The global inconsistency model was used to assess the consistency of the entire network and was considered good at p > 0.05 (12). A loop-specific approach was used to assess the presence of local inconsistencies in each closed loop. The node splitting method was used to assess the inconsistency of the model with separating evidence on a particular comparison into direct and indirect evidence (13). Funnel plots were plotted to evaluate the presence of publication bias.

## RESULTS

### Study selection and basic characteristics

**Figure 1:**
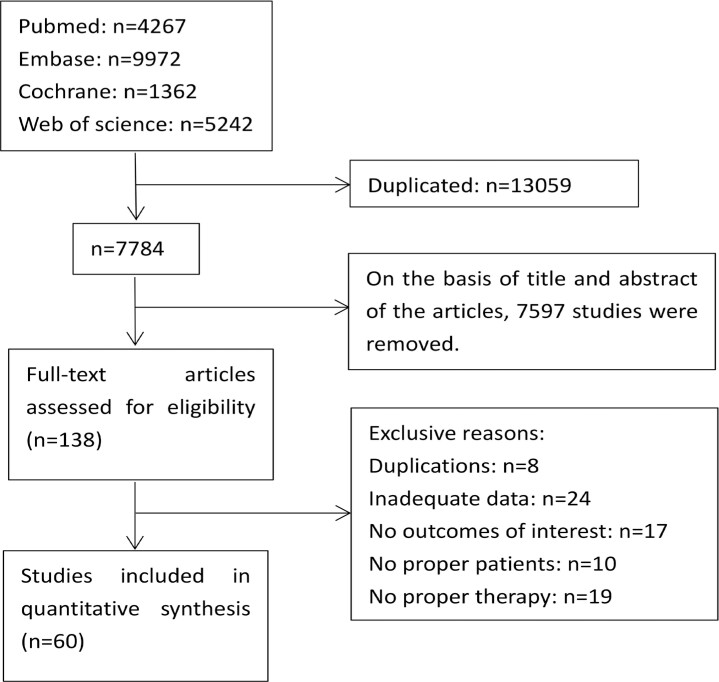
Flow Chart of Literature Search and Screening.

Through systematic search, 60 randomized, controlled, double-blind studies involving a total of 50,333 subjects were finally included. The literature search and screening process is shown in Figure-1, and the basic characteristics of the included studies are shown in Table-1.

### Evaluation of the quality of the included studies’ literature

A total of 60 randomized, controlled, double-blind studies were included, including 7 four-arm studies, 18 three-arm studies and 35 double-arm studies. The overall risk of bias was generally low. The risk of bias was assessed as shown in Supplementary Table-2 (see Page 2).

### Effectiveness indicators

### Mean daily micturitions

**Figure 2:**
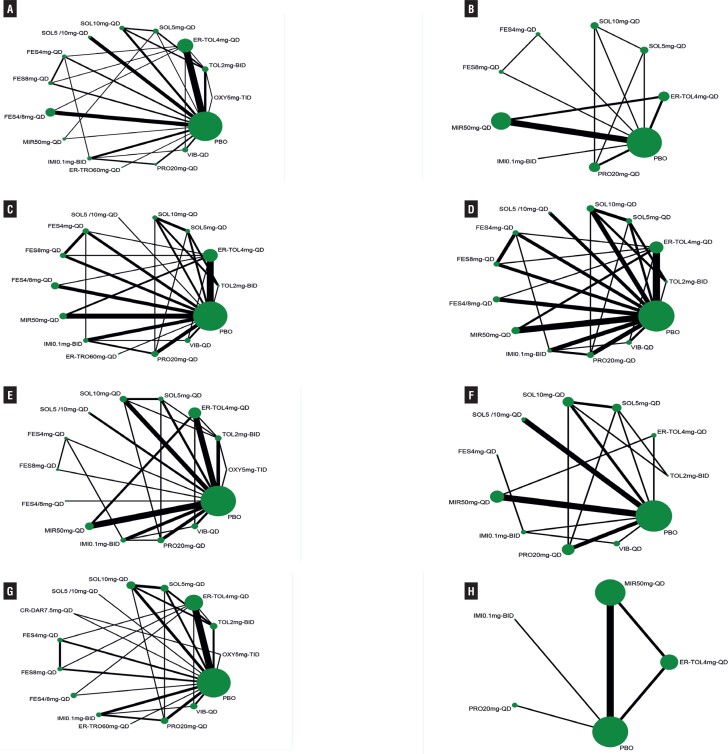
Evidence Network Plot for Micturitions with Female Proportion>50% (A), Micturitions with Female Proportion≤50% (B), Incontinence (C), Urgency (D), Urgency Incontinence (E), Nocturia (F), Voided Volume/micturition with Female Proportion>50% (G), Voided Volume/micturition. with Female Proportion ≤ 50%. Lines connect the interventions that have been studied in head-to-head (direct) comparisons in the eligible randomized controlled trials. The width of the lines represents the cumulative number of randomized controlled trials for each pairwise comparison, and the size of every node is proportional to the number of randomized participants (sample size).

**Figure 3:**
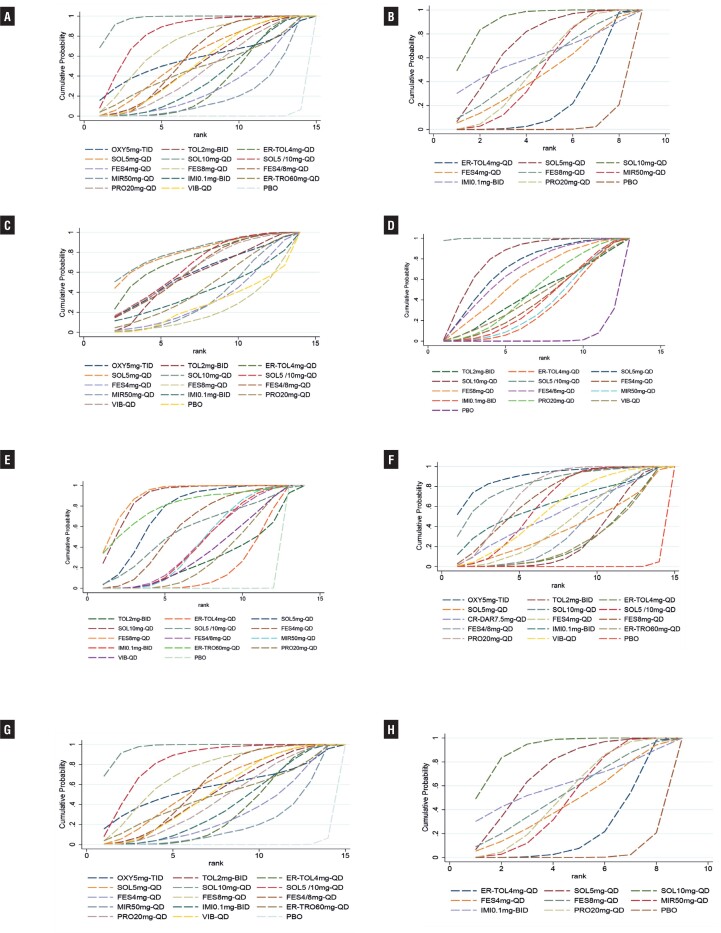
SUCRA Plot for Micturitions with Female Proportion>50% (A), Micturitions with Female Proportion≤50% (B), Incontinence (C), Urgency (D), Urgency Incontinence (E), Nocturia (F), Voided Volume/micturition with Female Proportion>50% (G), Voided Volume/micturition with Female Proportion ≤ 50% (H). (SUCRA: surface under the cumulative ranking curve. The larger the surface area, the higher the ranking).

Forty-two RCTs (14–56) reported micturition's/d, including 2 studies in 4 arms, 12 studies in 3 arms and 30 studies in double arms, containing a total of 15 treatment measures and a total sample size of 32,317 cases (Figure-2). Initial overall inconsistency testing showed a p-value <0.05 and partial p-values <0.05 in ring inconsistency, so subgroup regression analysis of the data according to the proportion of female patients showed that all inconsistency testing p-values were >0.05. For the subgroup with ≥50% female, all interventions were significantly more effective than placebo compared to placebo, except for oxybutynin (OXY)5mg-TID, with SOL10mg-QD being the most effective and significantly better than the majority of interventions. For the subgroup with less than 50% women, SOL10mg-QD remained the most effective, with statistically significant differences in efficacy compared to propiverine (PRO) 20mg-QD, mirabegron (MIR) 50mg-QD, extended-release tolterodine (ER-TOL) 4mg-QD and PBO. Results of the NMA are reported in Supplementary Table-3 (see Page 5). Figure-3 shows the mean values of SUCRA for interventions on micturitions.

### Mean daily incontinence episodes

Twenty-three RCTs (14–16, 25–44) reported incontinence episodes/d, including two 4-arm studies, eight 3-arm studies and 14 two-arm studies, comprising a total of 14 treatment measures and a total sample size of 15,632 cases (Figure-2). Among these studies, since the inclusion criteria for the Dmochowski 2003 et al. (54). study was “patients at least 18 years of age taking current pharmacologic treatment for OAB”, this study had significant clinical heterogeneity with other study populations, and the data were analyzed after excluding this study. The results showed that SOL10mg-QD was the most effective, followed by SOL5mg-QD and SOL5/10mg-QD. Results of the NMA are reported in Supplementary Table-4 (see Page 7). Figure-3 shows the mean values of SUCRA for interventions on micturitions.

### Mean daily urgency episodes

Thirty-one RCTs (15–20, 23–34, 37–49) reported urgency episodes/d, including three 4-arm studies, nine 3-arm studies and 19 two-arm studies, containing a total of 13 treatment interventions and a total sample size of 23,764 cases (Figure-2). The results suggested that SOL5 /10mg-QD was significantly more effective than other interventions in reducing the number of urinary urgency episodes, followed by SOL10mg-QD and SOL5mg-QD; while compared to placebo, TOL2mg-BID, VIB-QD, fesoterodine (FES) 4mg-QD, imidafenacin (IMI) 0.1mg-BID, MIR50mg-QD and ER- TOL4mg-QD's efficacy was improved, but the difference was not statistically significant. Results of the NMA are reported in Supplementary Table-5 (see Page 9). Figure-3 shows the mean values of SUCRA for interventions on micturitions.

### Mean daily urgency incontinence episodes

Twenty-nine RCTs (15–19, 22–30, 37–51) reported urgency episodes/d, including three 4-arm studies, eight 3-arm studies and 18 two-arm studies, containing a total of 14 treatment measures and a total sample size of 17,859 cases (Figure-2). The results showed that FES8mg-QD was the most effective in reducing mean daily urgency incontinence episodes, followed by SOL10mg-QD, with no statistically significant difference between the two, but both showed significant improvements in efficacy compared to most other interventions. FES8mg-QD was significantly more effective than FES4mg-QD and FES4/8mg-QD; while the difference in efficacy between SOL10mg-QD and SOL5mg-QD and SOL5/10mg-QD was not statistically significant. All interventions were significantly more effective than placebo and the differences were statistically significant, except for TOL2mg-BID which showed no significant improvement in efficacy differences compared to placebo. Results of the NMA are reported in Supplementary Table-6 (see Page 11). Figure-3 shows the mean values of SUCRA for interventions on micturitions.

### Mean daily nocturia episodes

Fifteen RCTs (17,18,24, 28–31,33, 37–42, 52) reported nocturia episodes/d, including one 4-arm study, three 3-arm studies and 12 two-arm studies, containing a total of 11 treatment interventions and a total sample size of 9,426 cases (Figure-2). The results showed that all interventions, except TOL2mg-BID, ER-TOL4mg-QD and FES4mg-QD, had significantly improved efficacy compared to placebo, and SOL5/10mg-QD had the best efficacy, followed by SOL10mg-QD and IMI0.1mg-BID. Results of the NMA are reported in Supplementary Table-7 (see Page 13). Figure-3 shows the mean values of SUCRA for interventions on nocturia.

### Voided volume per micturition

Twenty-seven RCTs (14–19, 22, 24–26, 29–31, 35–38, 40–44, 46–48, 53, 54) reported voided volume per micturition, including three 4-arm studies, ten 3-arm studies, and fourteen two-arm studies containing 11 treatment measures with a total sample size of 9,426 cases (Figure-2). Initially, subgroup regression analysis was performed due to inconsistencies. The results showed a global inconsistency of p-value > 0.05 after subgroup analysis according to the percentage of females. In the subgroup with ≥ 50% female, OXY5mg-TID had the best efficacy, followed by SOL10mg-QD and PRO20mg-QD, a result consistent with the initial overall results. In the subgroup with < 50% female representation, ER-TOL4mg-QD, MIR50mg-QD and PRO20mg-QD were significantly more efficacious than the placebo group, with only the IMI0.1 mg-BID group shared no significant difference with the placebo group. In contrast, compared to the placebo, IMI0.1mg-BID in the subgroup with ≥ 50% female and the initial overall outcome posed a significant difference in efficacy. Results of the NMA are reported in Supplementary Table-8 (see Page 15). Figure-3 shows the mean values of SUCRA for interventions on voided volume per micturition.

### Safety outcomes

**Figure 4:**
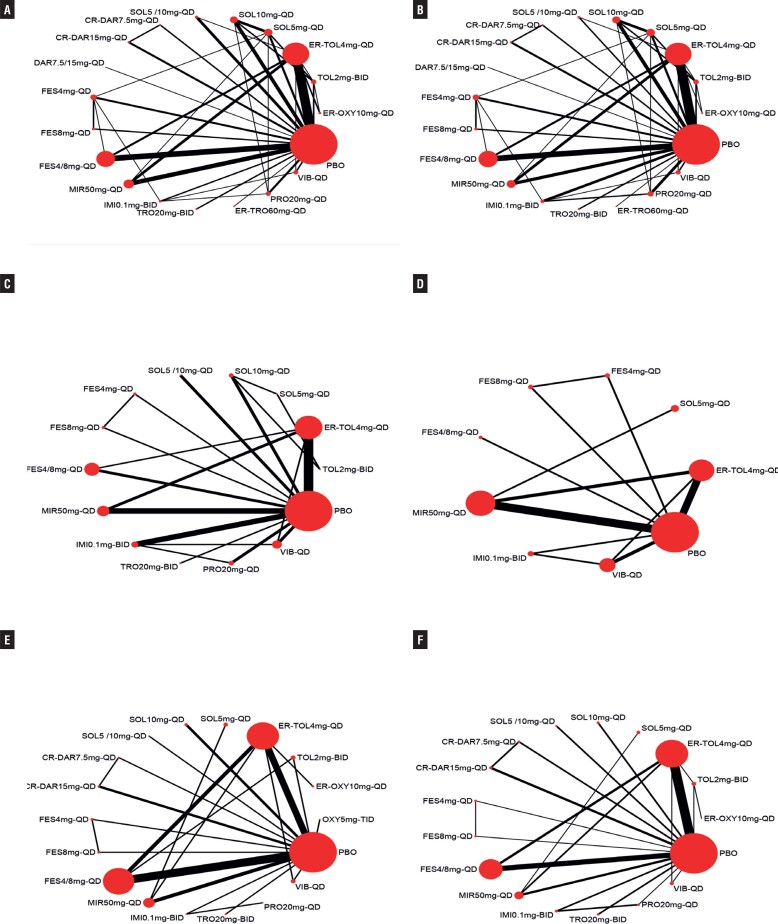
Evidence Network Plot for Dry Mouth (A), Constipation (B), Nasopharyngitis (C), Hypertension (D), Urinary Tract Infection (E), Headache (F). Lines connect the interventions that have been studied in head-to-head (direct) comparisons in the eligible randomized controlled trials. The width of the lines represents the cumulative number of randomized controlled trials for each pairwise comparison, and the size of every node is proportional to the number of randomized participants (sample size).

**Figure 5:**
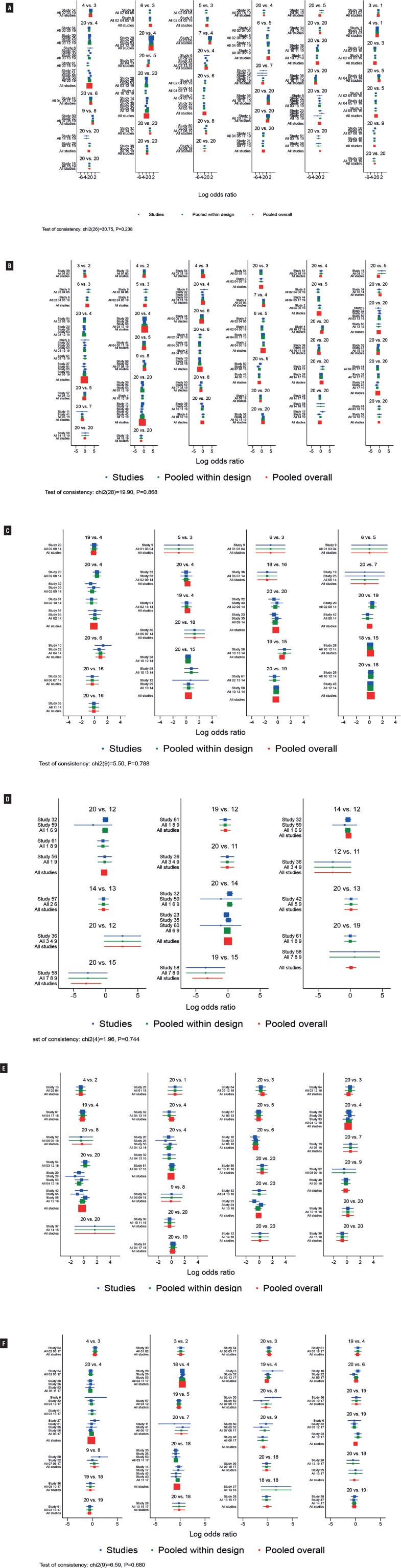
NMA Forest Plot for Dry Mouth (A), Constipation (B), Nasopharyngitis (C), Hypertension (D), Urinary Tract Infection (E), Headache (F). (The consistency of the entire network and was considered good at p > 0.05).

**Figure 6:**
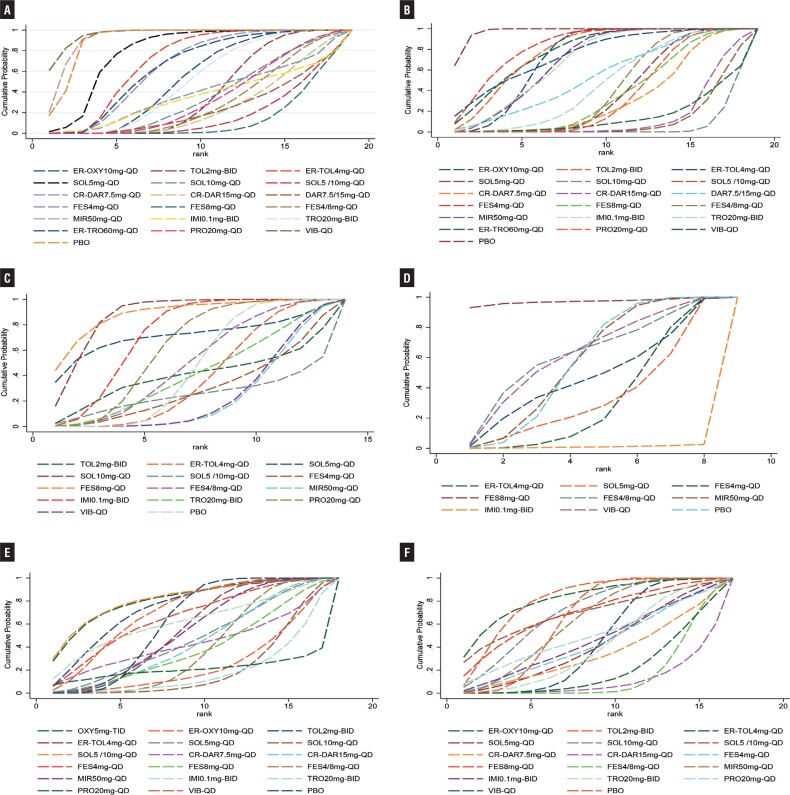
SUCRA Plot for Dry Mouth (A), Constipation (B), Nasopharyngitis (C), Hypertension (D), Urinary Tract Infection (E), Headache (F). (SUCRA: surface under the cumulative ranking curve. The larger the surface area, the higher the ranking).

Fifty-five RCTs (14, 16–18, 20–31, 33–41, 43–53, 56–73) reported dry mouth, and to exclude nocebo effect on study outcomes, two articles (14, 25) with significantly higher data in the placebo group than in other studies were excluded. Therefore, two 4-arm studies, 17 three-arm studies, and 34 two-arm studies, containing a total of 19 treatment measures and a total sample size of 45,756 cases, were considered (Figure-4). The results showed that the interventions with the lowest incidence of dry mouth were VIB-QD, MIR50mg-QD and PBO respectively. Constipation was reported in 50 RCTs, including two 4-arm studies, 18 three-arm studies, and 30 two-arm studies, containing a total of 19 treatment measures and a total sample size of 45,674 cases. The incidence of constipation was not significantly higher for FES4mg-QD, ER-OXY10mg-QD, TOL2mg-BID, and VIB-QD compared with placebo, while the incidence of constipation was higher for the remaining interventions than for the placebo group. A total of nine interventions were included for hypertension, of which only IMI0.1 mg-BID caused a significant difference in the incidence of hypertension compared with placebo and other treatments, and the remaining seven were not significantly different compared with placebo. For headache, 17 interventions were included, and only FES4/8mg-QD and CR-DAR15mg-QD were found to exhibit a significantly higher incidence compared to placebo. A total of 18 interventions were included for urinary tract infections, and their incidence with only SOL10mg-QD differed statistically significantly from placebo. Figure-5 shows the mean values of SUCRA for interventions on AEs. Results of the NMA are reported in Supplementary Tables 9-14 (see Page 17-30). Figure-6 shows the mean values of SUCRA for interventions on safety outcomes.

### Inconsistency and heterogeneity check

**Figure 7:**
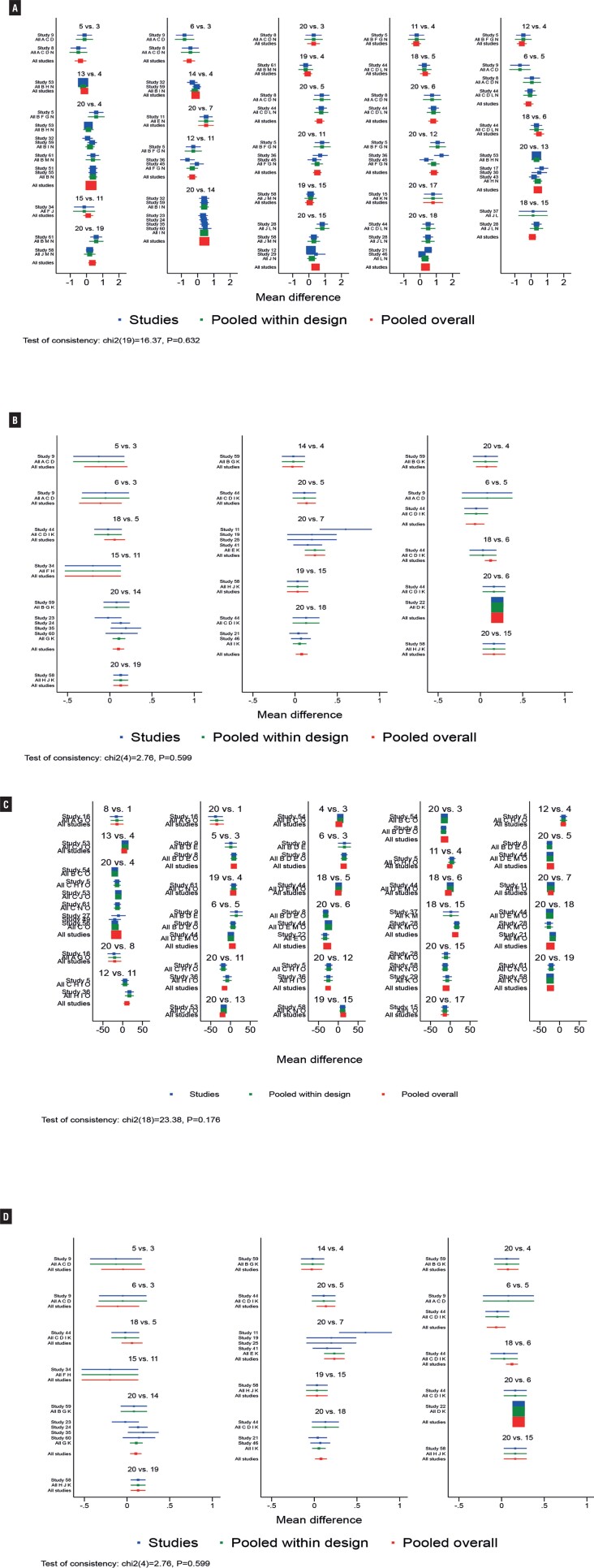
NMA Forest Plot for Urgency Incontinence (A), Nocturia (B), Voided Volume/micturition with Female Proportion>50% (C), Voided Volume/micturition with Female Proportion≤50% (D). (The consistency of the entire network and was considered good at p > 0.05.)

**Figure 8:**
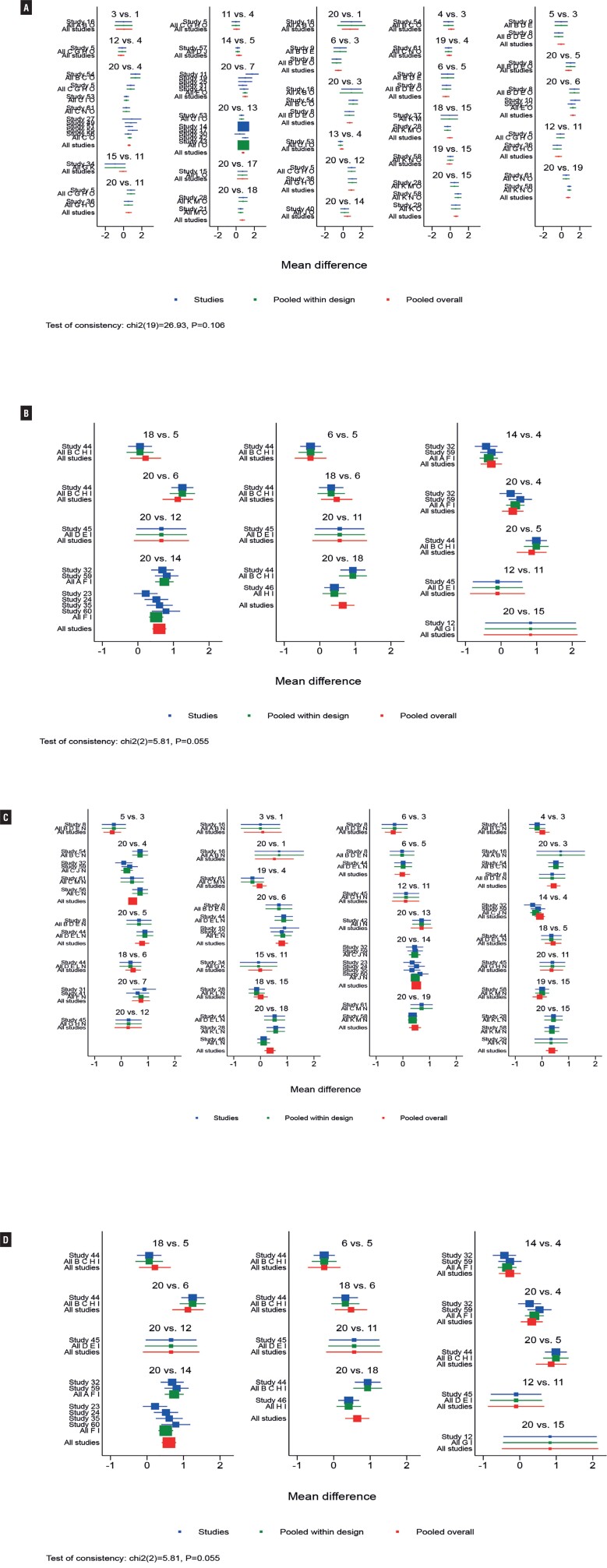
NMA Forest Plot for Micturitions with Female Proportion>50% (A), Micturitions with Female Proportion≤50% (B), Incontinence (C), Urgency (D). (The consistency of the entire network and was considered good at p > 0.05.)

Initially, in improving mean daily micturition's and voided volume per micturition, overall inconsistency testing showed inconsistency (P value < 0.05) and inconsistency in individual rings (95% CIs not including 1), and subgroup analysis based on race, duration of disease, and other factors did not reveal significant improvement. Therefore, subgroup regression analysis of the data according to the proportion of female patients showed that the overall inconsistency and ring inconsistency p values were >0.05. In terms of reducing mean daily incontinence episodes, sensitivity analysis showed that the study by Dmochowski 2003 et al. (54). was significantly different from other studies, considering that the inclusion criteria for the study were “patients at least 18 years of age taking current pharmacologic treatment for OAB”. Therefore, this study showed significant clinical heterogeneity with other study populations in the efficacy index of reduction in the number of incontinence episodes. Thus, analysis of the data upon excluding this study would show no inconsistency. The global inconsistency model showed well with p>0.05 (Figures 6-8). The result of local inconsistency showed that most loops were consistent according to the 95%CI. The test for inconsistency using node-splitting model revealed no significant difference between direct and indirect comparisons (P>0.05).

### Publication bias

**Figure 9:**
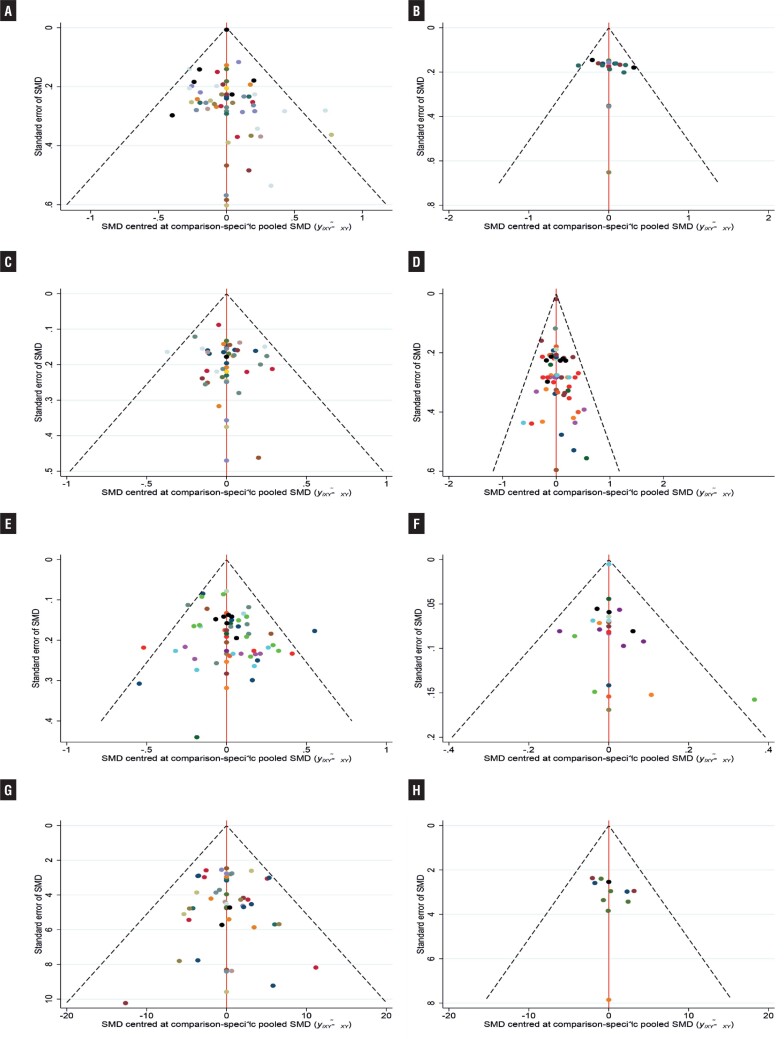
Funnel Plot for Micturitions with Female Proportion>50% (A), Micturitions with Female Proportion ≤ 50% (B), Incontinence (C), Urgency (D), Urgency Incontinence (E), Nocturia (F), Voided Volume/micturition with Female Proportion>50% (G), Voided Volume/micturition with Female Proportion ≤ 50% (H). (The distribution of each point in the funnel plot is roughly symmetrical, suggesting that there is no publication bias or other bias in the studies).

A funnel plot was established to assess the publication bias. There was no significant evidence of publication bias for outcomes based on a Begg funnel plot (Figure-9).

## DISCUSSION

OAB is a chronic syndrome that is not life-threatening and does not progress to uncontrollable functional impairment but has serious impacts on the patient's quality of life. Therefore, current research is increasingly focused on the impact of interventions on the quality of life of patients with OAB. For OAB treatment, improving patients’ symptoms and reducing the incidence of adverse events are equally important for improving patients’ quality of life and treatment compliance. This study aims to compare the therapeutic effects of different interventions in terms of efficacy and safety, and to identify the advantages and disadvantages of different drugs in the process of clinical application, so as to provide more direct data support for the individualized treatment and drug use of different patients in the clinic.

Ten OAB therapeutic agents were included in this study, involving a total of 19 interventions grouped by different doses administered, and the NMA results show that solifenacin had a relatively good overall efficacy and a significant advantage in improving patients’ symptoms. Solifenacin 10mg was the most effective in reducing the number of voiding and incontinence; solifenacin 5/10mg was the most effective in reducing urinary urgency and nocturia; solifenacin 10mg ranked second in both urgency incontinence and voided volume. In terms of safety, the incidence of dry mouth events with solifenacin 5mg was not significantly different from placebo and was significantly lower than other anticholinergic drugs. Solifenacin is a competitive antagonist of M3 receptors and is highly specific and selective for bladder M3 receptors. The results of past studies have shown that solifenacin has a weaker blocking effect on salivary secretion than other anticholinergic drugs and that it inhibits salivary secretion at 3.6-6.5 times the effective concentration at which it produces an effect in the bladder (74, 75), which is consistent with the results of the present study. However, in the case of constipation, the results of this study showed that even a small dose of solifenacin (5mg) increased the incidence of constipation. Constipation has the greatest effect on patient satisfaction (76). Therefore, the results suggest that solifenacin is not recommended for the clinical treatment of patients with OAB who are prone to constipation.

Different interventions have different pharmacological characteristics, and different doses may affect the efficacy of treatment, in addition to their safety. Therefore, it is necessary to select the appropriate medication and dose according to the individual patient's condition so that the patient's quality of life can be maximized. This NMA analyzed the incidence of dry mouth, constipation, nasopharyngitis, headache, hypertension, and urinary tract infection in the included studies and showed that anticholinergic drugs may increase the incidence of dry mouth and constipation, while imidafenacin may increase the risk of hypertension, and FES4/8mg-QD and CR-DAR15mg-QD increase the incidence of headache compared to placebo. SOL10mg-QD may increase the risk of urinary tract infections.

Before choosing a treatment plan, the benefits of the treatment plan for the patient and the possible risks and complications should be fully considered, and decisions should be made after weighing the pros and cons. In terms of efficacy, vibegron and mirabegron are superior to placebo and comparable to anticholinergics; although they do not show an efficacy advantage over anticholinergic drugs, their greatest advantage is in terms of safety, with both drugs showing good tolerability. In particular, vibegron and mirabegron have a significant advantage over cholinergic receptor antagonists with respect to dry mouth. As potent β3 agonists, vibegron and mirabegron relax the detrusor muscle by activating β3 receptors, thereby increasing bladder capacity and prolonging the interval between voiding without affecting bladder voiding activity. The selectivity for β3 receptors over other β receptor subtypes also suggests that both drugs are effective and well-tolerated novel drugs for OAB patients (77, 78).

In the voided volume per micturition outcome indicator, there was inconsistency between the direct and indirect comparison results of SOL10mg-QD and PRO20mg-QD (p-value 0.017). Although the direct and indirect comparisons were significantly different, the results of the two interventions compared pointed towards the same direction, suggesting that SOL10 mg-QD was superior to PRO20 mg-QD, varying only in the degree of their difference, so the results were considered to be somewhat reliable.

Because of the overall inconsistency in this NMA study in terms of decreasing micturition/d and increasing voided volume/micturition, a subgroup regression analysis was performed. Despite the differences between male and female in the anatomy and physiology of the lower urinary tract system and the potential mechanisms of action that may lead to OAB-like symptoms (79), none of the clinical studies included “gender” as an analyzable data in detail, but simply expressed whether the proportion of women was ≥50%, so only subgroups of women ≥ and <50% were analyzed in this study. The results of the subgroup analysis suggest that the results of imidafenacin are opposite in the subgroups with greater than and less than 50% women, so it is speculated that the efficacy of imidafenacin in men and women may vary, which would need to be confirmed by the results of more single-sex studies.

To control for homogeneity in the included studies, strict entry row criteria were established, and all 12-week efficacy indicators were used as the endpoints examined in this study, which avoided the introduction of clinical heterogeneity due to different study periods. Some limitations still exist in this study: 1. Because the quality of life measurements used in different studies are not uniform, this indicator of quality of life has not been analyzed and compared. Clinical endpoints can assess the effectiveness of symptom treatment from an objective perspective, but further research is needed to determine whether these symptom changes are relevant to the improvement of patients’ quality of life. 2. No subgroup analysis of age was performed in this study. Existing studies have shown differences in the effectiveness of solifenacin versus mirabegron in elderly and non-differentiated age groups (80). However, only 2 of the studies included in this study enrolled elderly subjects, so subgroup analysis could not be performed. 3. No comparative study of long-term medication use was conducted in this study. Overactive bladder requires long-term medication treatment, and the data from the 12-week study used in this study are not representative of its true efficacy and safety.

## CONCLUSIONS

Individualized treatment based on the characteristics of the patient is crucial. Anticholinergic drugs carry a risk of increased incidence of dry mouth and constipation, with lower doses carrying a lower risk. Solifenacin (10mg, 5mg/10mg) has significant advantages in improving patient symptoms. However, even low doses of solifenacin (5mg) can increase the incidence of constipation. In addition, imidafenacin may increase the risk of hypertension, FES4/8mg and CR-DAR15mg may increase the incidence of headaches, and SOL10mg-QD may increase the risk of urinary tract infections. These drugs should be used with caution in patients at risk for these side effects. Although the efficacy of mirabegron and vibegron is not superior to anticholinergic drugs, they are better tolerated by OAB patients.
